# Project design and technology trade-offs for implementing a large-scale sexual and reproductive health mHealth intervention: Lessons from Sierra Leone

**DOI:** 10.3389/fdgth.2023.1060376

**Published:** 2023-03-13

**Authors:** Emeka Chukwu, Sonia Gilroy, Kim Eva Dickson

**Affiliations:** United Nations Population Fund (UNFPA) Country Office, Freetown, Sierra Leone

**Keywords:** text message, interactive voice response (IVR), radio, mHealth, digital health (eHealth)

## Abstract

**Background:**

The Coronavirus 2019 (COVID-19) pandemic threatened decades of progress in sexual and reproductive health (SRH) and gender-based violence as attendance at health facilities plummeted and service uptake dwindled. Similarly, misinformation regarding COVID-19 was rife. The demographics in Sierra Leone are diverse in the education, economic, and rural/urban divide. Telecommunications coverage, phone ownership, and preference for information access medium also vary greatly in Sierra Leone.

**Aim:**

The aim of the intervention was to reach Sierra Leoneans at scale with information about SRH during the early stages of the COVID-19 pandemic. This paper presents the approach and insights from designing and implementing a large-scale mobile health (mHealth) messaging campaign.

**Method:**

Between April and July 2020, a cross-sectional multichannel SRH messaging campaign was designed and launched in Sierra Leone. Through a secondary analysis of project implementation documents and process evaluation of the messaging campaign report, the project design trade-offs and contextual factors for success were identified and documented.

**Result:**

A total of 1.16 million recorded calls were initiated and 35.46 million text messages (short message service, SMS) were sent to telecommunication subscribers through a two-phased campaign. In phase one, only 31% of the 1,093,606 automated calls to 290,000 subscribers were picked up, dropping significantly at 95% confidence level (*p* = 1) after each of the four weeks. In addition, the listening duration dropped by one-third when a message was repeated compared to the first 3 weeks. Lessons from phase one were used to design an SMS and radio campaign in the scale-up phase. Evidence from our analysis suggests that the successful scaling of mHealth interventions during a pandemic will benefit from formative research and depend on at least six factors, including the following: (1) the delivery channels’ selection strategy; (2) content development and scheduling; (3) the persona categorization of youths; (4) stakeholder collaboration strategies; (5) technology trade-offs; and (6) cost considerations.

**Discussion and Conclusion:**

The design and implementation of a large-scale messaging campaign is a complex endeavor that requires research, collaboration with other diverse stakeholders, and careful planning. Key success ingredients are the number of messages to be delivered, the format, cost considerations, and whether engagement is necessary. Lessons for similar low-and-middle-income countries are discussed.

## Background

Sexual and reproductive health (SRH) organizations continue to search for efficient and effective ways to reach young people in this age of mobile technologies (mHealth). Traditional health education strategies are becoming obsolete for the new generation of young people. There are 79 mobile phone subscribers per 100 individuals in Sierra Leone, representing 6.3 million Sierra Leoneans (1, 2). However, phone ownership remains at 36 mobile phones per 100 individuals, meaning 2.87 million people (1). While this teledensity may be lower than in many places around the world, it still presents the opportunity for a wider reach at a relatively lower overhead. Only 14.3% of subscribers use the 100-MB internet group bracket per month, and only 7.1% use 1 GB and above nternet data every month (1). In addition, Facebook, one of the most dominant social media platforms in the country, has approximately 700,000 users (3). WhatsApp is also popular in Sierra Leone among all age groups but predominantly among millennials. Policymakers and international audiences interested in national policy directions also follow updates through Twitter. Stakeholders in Sierra Leone have deployed many ongoing digital-enabled interventions, mostly facility-based applications (4, 5). The search of mHealth and Sierra Leone in the PubMed database did not yield any client-facing mHealth intervention. This project is the first documented mHealth intervention, and also one of the leading interventions at this scale globally.

The Coronavirus 2019 (COVID-19) pandemic did not only put a strain on the already weak health system in Sierra Leone, but its fear also impacted service uptake and attendance at general health facilities. During the lockdown period that followed, there was a visible surge in cases of gender-based violence (GBV). Mobile technologies have been used to successfully deliver SRH messages to subscribers in Africa (6–9), Asia (10, 11), and the United States (12). Some campaigns targeted recipients through social media channels in China (13), Turkey (14), and Hong Kong (15). An interactive voice response (IVR) mobile content delivery channel has been used for maternal health (MH) (16), post-abortion care (11), and family planning (FP) (6). IVR-delivered contents were mostly in the native local language (not in English). Short message service (SMS) interventions for family planning helped improve consumer knowledge by 14% (8). Social media has also been used in other regions for reproductive health-based demand generation, like China's peer-led safe sex Facebook group (13). Other demand generation interventions include serious games to enhance sex education for young adolescents in Hong Kong (17). Serious games use virtual reality-enabled games with engaging family planning information. A video-based mobile technology intervention has equally shown promise among adolescents in the United States (18). In Kenya, the Shujaaz multimedia platform used various channels ranging from comic radio programs, Facebook campaigns, and SMS (19).

The United Nations Populations Fund (UNFPA), through the Saving Lives project, launched a multichannel intervention project between April and July 2020 to reach young people in Sierra Leone with critical SRH messages using mobile technology. The project involved the design and deployment of the interventions through multiple messaging channels. Multichannel here refers to the use of several media channels for spreading marketing and health promotion and education messages to consumers and service users, including *via* social media, print, mobile, television, etc. (20, 21). The multiple channels adopted in this project were automated voice calls, SMS, radio jingles, and social media. The campaign followed earlier formative research studies with school counselors, community learning centers, and youth advisory panels (20). The campaign helped to communicate the following: continuity of sexual reproductive health; how to seek help for gender-based-violence-related issues; and staying safe during the COVID-19 restrictions and countermeasures. We identified the initial target and priority population as Freetown residents. As of situation report 30, Freetown, the main urban center in Sierra Leone, had the highest number of COVID-19 infections (84 of the national 116) and cumulative deaths (all five deaths) (22). The intervention was later scaled nationwide.

Sierra Leone has three major telecommunications service providers: Africell, Orange, and Q-cell. Our intervention worked with Africell because they responded positively to our call for partners and they had the widest audience reach in the country. We targeted all their approximately 3.9 million subscribers out of the national total of 6.9 million mobile subscribers (3).

The objective of the messaging campaign was to create awareness of the continuity of GBV and SRH services during COVID-19 restrictions. The aim of the present paper is to show the design choices, approach, and strategies for the pilot phase and for the nationwide scale-up phase of the intervention. The paper also discusses the messaging campaign's call pickup rates, duration of listening, overall effectiveness, and other lessons learned for future similar interventions. The lessons learned were also documented from designing and implementing the multi-intervention project in Sierra Leone using the framework from Allsop et al. (23).

## Materials and methodology

The approach for designing and the deployment of the multichannel intervention is first presented, followed by the evaluation. The intervention components were discussed, including content adaptation, transcription, recording, channel selection, message scheduling, and targeting. The internal document and report reviews and evaluation were conducted after the intervention to extract learnings for future interventions.

### The intervention approach

#### Intervention setting

Sierra Leone covers a land area of 72,180 km^2^ with an estimated population of 8.4 million people (2, 24). Based on the projections of the latest United Nations data, 43% of the population resides in urban areas (2). Freetown, the capital city, is the main urban district with a population of 802,639, split between the western urban and western rural areas. The country is made up of 14 health districts. Sierra Leone is one of the least developed countries in the world. Access to healthcare is limited by the inequitable allocation of skilled healthcare workers, poor service quality, geographical barriers, and high out-of-pocket expenses for health (24). Moreover, health facilities are unevenly distributed, with referral hospitals concentrated in Freetown (25).

#### Intervention formative research

**Figure 1:**
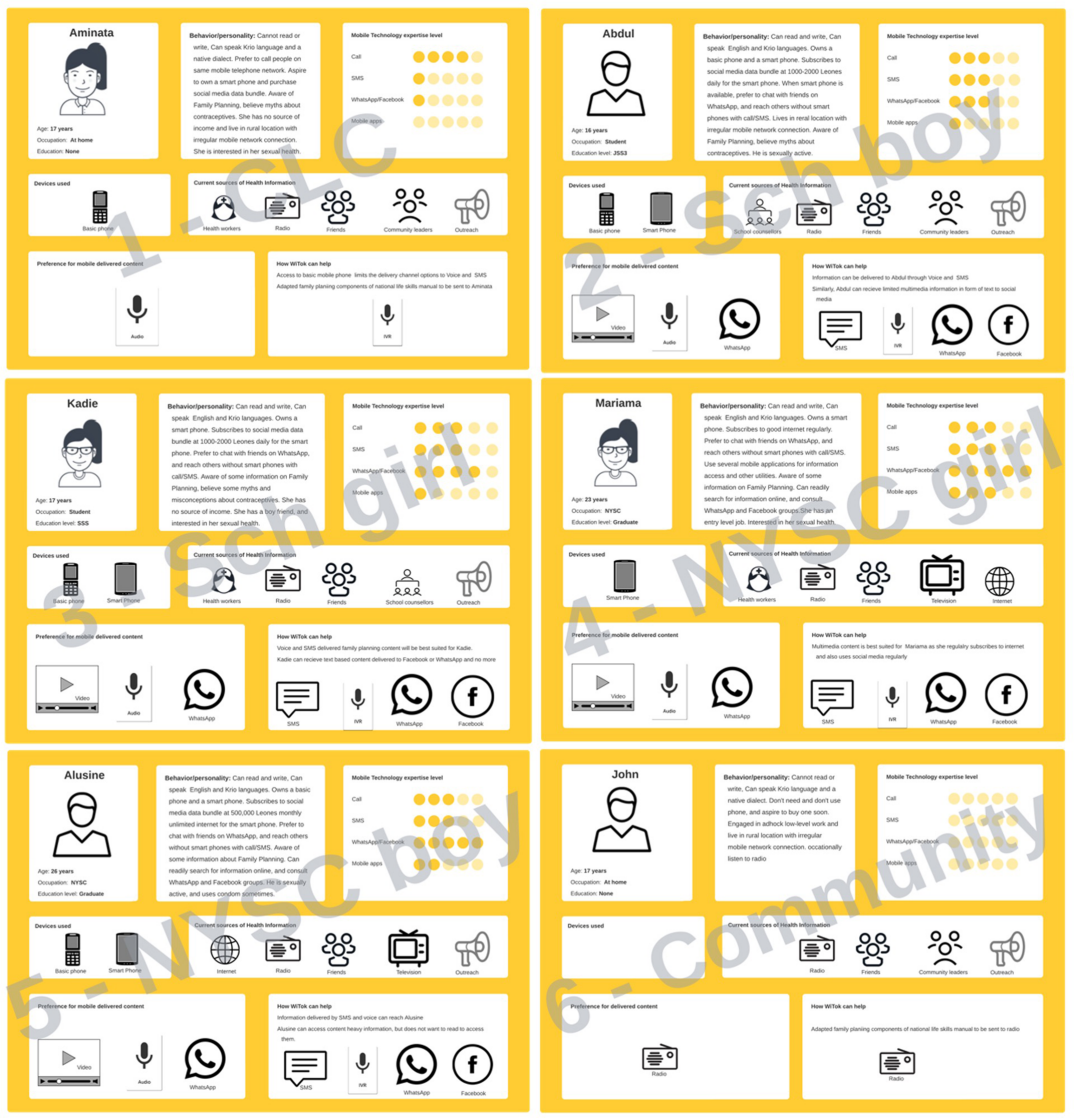
Six user personas developed as part of the UNFPA WiTok intervention approach. UNFPA, United Nations Populations Fund.

In 2019, UNFPA conducted formative research to understand how young people use mobile phones and the best strategy for reaching young people using mobile technology with SRH messages. The report showed that phone ownership and phone type increased with education and income (20). Young people, out of school, generally used basic phones and often could not read or write. Educated young people in secondary school used a mix of smartphones and basic mobile phones and were able to read and write. Graduates generally had smartphones and were comfortable using social media. User personas were developed for the six groups of participants, as in Figure 1. The personas show the demographic information, behavior/personality/lifestyle, their level of phone use expertise, current source of sexual reproductive health information, their preferred information source, and how the intervention can potentially help. The following paragraphs describe the multiple channels for spreading SRH messages. From the Leeds EPaCCS program evaluation hierarchy, it was determined that the published formative research addressed the pre-implementation usability and technical aspects (23).

#### Intervention content adaptation, translation, and recording

The UNFPA Sierra Leone country office constituted an internal multidisciplinary task team to facilitate the development and adaptation of SRH and GBV content for delivery to Sierra Leoneans. The team included technical members specializing in maternal health, family planning, gender, communications, Monitoring and Evaluation (M&E), the audiovisual, and mHealth focal points. Furthermore, the team partnered with other UN agencies (UN Women and UNICEF) and government ministries (Ministries of Gender and Children's Affairs, and Health and Sanitation) to develop, review, and approve the messages. The team met regularly in person and later adopted WhatsApp group collaboration coupled with Zoom calls due to the COVID-19 measures. The team developed relevant messages through several brainstorming sessions around three thematic areas in line with the UNFPA core mandate, namely, GBV, FP, MH, and COVID-19. Select contents were agreed to and first developed into English master text transcripts through an iterative review process. The master transcripts were then interpreted and transcribed into the Krio language because Krio is widely spoken in Sierra Leone. We also back-translated to ensure the adequacy of the translation. The Krio messages were audio-recorded for radio and automated voice calls. The recorded messages had a 1-min restriction and were recorded in a jingle style. The English transcript master file was also used to adapt SMS format messages with the 160-character limit.

The number of unique messages developed by the thematic area is shown in Table 1.

**Table 1:** Number of developed messages by thematic area.

Thematic area	Audio (Radio)	Audio (auto-calls)	Text (SMS)	Audiovisual (Social media)
GBV	6	1	6	6
MH	4	1	4	4
FP	2	1	2	2
COVID-19	1	0	1	1

SMS, short message service; GBV, gender-based violence; MH, maternal health; FP, family planning; COVID-19, coronavirus 2019.

#### Intervention content delivery channels

##### Social media

The channels identified for the delivery of social media messages were WhatsApp groups and the UNFPA Sierra Leone Twitter account. Each message was shared every Monday, Wednesday, and Friday. On designated days, the first message was shared *via* UNFPA Twitter, and additional messages could be shared further thereafter. Ten team members provided a total of 39 WhatsApp groups, each with 200–250 members in Sierra Leone, where they recommended sharing the WhatsApp content. The messages were also shared through the UNFPA Facebook page on the same days. The content delivery strategy was to ensure alignment with other channels.

##### Radio

Based on advice from the communications analyst, four radio stations were needed to cover the entire Freetown area. Freetown was chosen because it is urban and with the highest number of COVID-19 infections in the country. Each of the four radio stations played a schedule of GBV, FP, MH, and COVID-19 messages in the mornings and evenings using the specified schedules.

##### Telecommunications

Messages were designed and delivered to telecommunications subscribers using different strategies throughout the life of the campaign. Initially, messages targeted UNICEF U-Reporters subscribers. Subsequently, Africell telecommunications subscribers were targeted based on their network data subscription with a pre-recorded automated voice call or SMS. Africell telecommunications is one of the three main subscribers in Sierra Leone. The messaging campaign was later scaled to a nationwide SMS campaign to all 3.9 million subscribers on the network.

##### Technology decisions and trade-offs for telecommunication messages

This technical section details the technology decisions and trade-offs for IVR, voice calls, and SMS. The trade-offs were based on the cost, willingness of the telecommunications service providers, technical capacity, and security considerations. As part of our engagement, the National Telecommunication Authority of Sierra Leone (NATCOM) indicated that mobile network operators (MNOs) do not support hardware-based gateways. Further engagement with the MNOs showed that they support software-based gateway solutions only and that the leading software GSM gateway is an open source technology called Asterisk and its other licensed derivatives. Because of this, the research undertaken in March and April focused on Asterisk being open source and known in the industry (almost the only solution), and it was the natural option to investigate and consider it (26).

###### 2080 UNICEF 2080 SMS short-code

The SMS channel has been used for SRH services with mixed success (27). Findings from our research from 2019 show that current leading SMS options for interactivity are limited to RapidPro (28) and Textit (29). They both have the same design, which includes an SMS flow designer and reporting interface. RapidPro is proprietary to UNICEF, and UNICEF has already configured the system in many of their UNICEF countries. The Textit application is licensed (proprietary) and available for anyone interested to acquire and use at a fee. They are both cloud-based services (i.e., hosted by a cloud service provider and not on UNFPA premises or Telecommunications premises). This takes away the need for hosting and server administration, as is the case for voice calls or IVR-based systems. The 67 FP messages developed in 2019 were designed into interactive flows and tested on the Textit SMS platform and uploaded on the U-Report SMS platform in November and December 2019.

**Figure 2:**
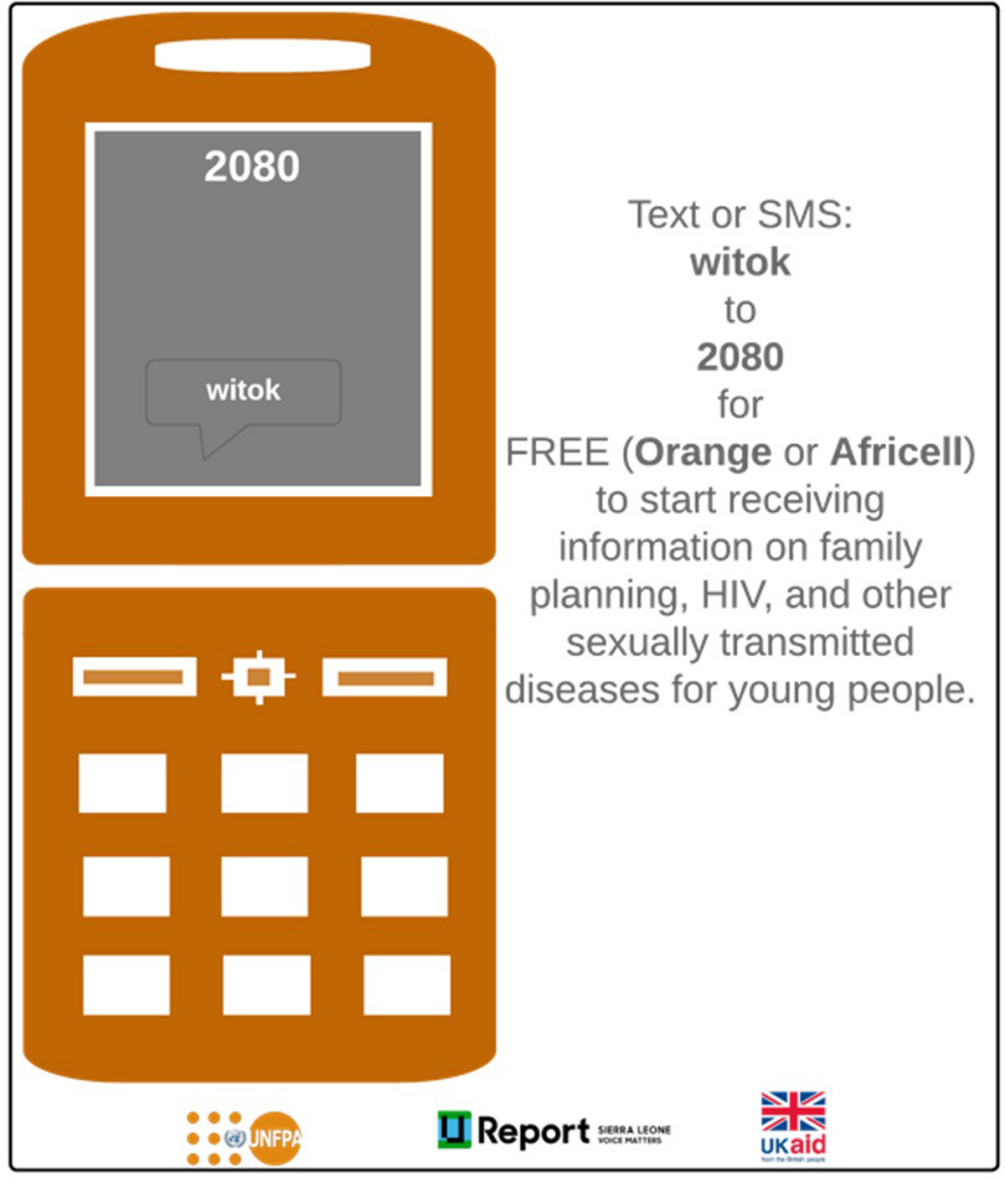
Proposed flyer for advertising the short code.

The message flow for the 67 SRH messages was configured on the UNICEF RapidPro platform. The main advantage of the RapidPro flow system is that it enables message personalization based on recipient engagement with the platform. The original plan was to advertise the intervention short code and allow users to opt in for in-depth SRH messages, as seen in the proposed flyer in Figure 2. The lack of dedicated staff with the requisite capacity to focus on the project resulted in little platform monitoring and content obsolescence. As a result, UNICEF disabled the controls after 30 days (starting in December 2019).

###### 2422 Africell 2422 SMS short-code

In addition, as part of a Memorandum of Understanding (MoU) between UNFPA and Africell, Africell delivered SMS content on behalf of UNFPA to registered subscribers through their own Africell SMS server platform. A decision was made to deliver the initial SMS messages using UNFPA as the ID instead of 2422, and this may change in the future. Content delivered on behalf of UNFPA by Africell was not interactive and were push-only-based systems. At the end of the campaign, Africell had delivered over 35.46 million FP, GBV, and MH messages nationwide using this approach. Africell emailed a monthly message delivery log with an aggregate bio-details distribution of recipients.

##### Voice messages

Voice messages have been shown to be effective in increased service uptake (30, 31). Technically, using Asterisk will require technical knowledge to manage the physical server hosting, its administration, and regular content updates. Server hosting can be either of three options: the internet cloud service providers (e.g., Google, AWS, or MS Azure); in the UNFPA office; or at the premises of the telecommunications service provider. The MoU with Africell ensured that Africell hosts and manages the message delivery with spare capacity on their server. A dedicated server managed by Africell would mean the added responsibility of server content updates, updating flow appearance, and assigning permissions to UNFPA.

The initial strategy was in three stages: first, to deliver recorded messages using the Africell existing system; second, to transition from an Africell temporary server to a UNFPA local server; and third, to evaluate the performance and throughput and choose the right server for the given scale desired. The first step of this process started as planned with Africell provisioning their server (spare capacity) with the UNFPA short code 2422 and delivering calls beginning 23 April 2020. However, the Africell system could not make the agreed 290,000 calls three times per week. The bottleneck meant that it took 1 week of daily calls at off-peak periods to complete the 290,000 calls (and). As a result, the efforts were discontinued after careful consideration of the technical trade-offs.

#### Intervention partner collaborations

**Figure 3:**
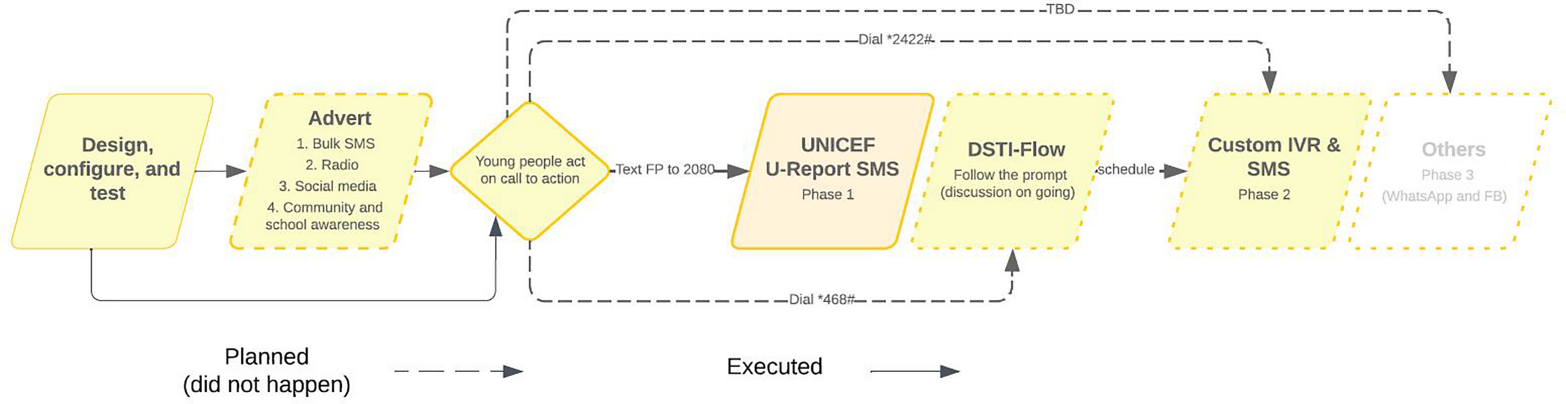
Conceptual strategy for working with collaborating partners.

The WHO and its partners have identified collaboration as critical for the success of mHealth interventions, particularly in low- and middle-income countries (32). The project was designed to reuse all existing systems from existing partners as much as possible. Stakeholder engagements were conducted with organizations that currently had an mHealth or SRH intervention or were planning one in the country. At the end of the conversation, two organizations were at the top of the collaboration and engagement list—UNICEF and the Directorate of Science, Technology and Innovation (DSTI) (33). At UNFPA, a conceptual strategy for engaging and working with both stakeholders was outlined, as in Figure 3. The Directorate of Science, Technology, and Innovation has just launched a multi-sectoral USSD platform (*468#) and was testing it (34). The engagement with UNFPA was to ensure integration and utilization for either messaging service enrollment or messaging service delivery to young people in Sierra Leone. Similarly, UNICEF has been operating the U-Report SMS platform for almost a decade in Sierra Leone. The aim of the engagement was to leverage the infrastructure, experience, and shared resources to bootstrap the project.

##### Collaboration with UNICEF

The RapidPro only supported SMS in Sierra Leone at the time of the project, though plans were ongoing to integrate other telecommunications channels such as USSD and Voice. The UNFPA Sierra Leone then opted to leverage the SMS infrastructure of RapidPro to deliver essential messages to young people.

**Figure 4:**
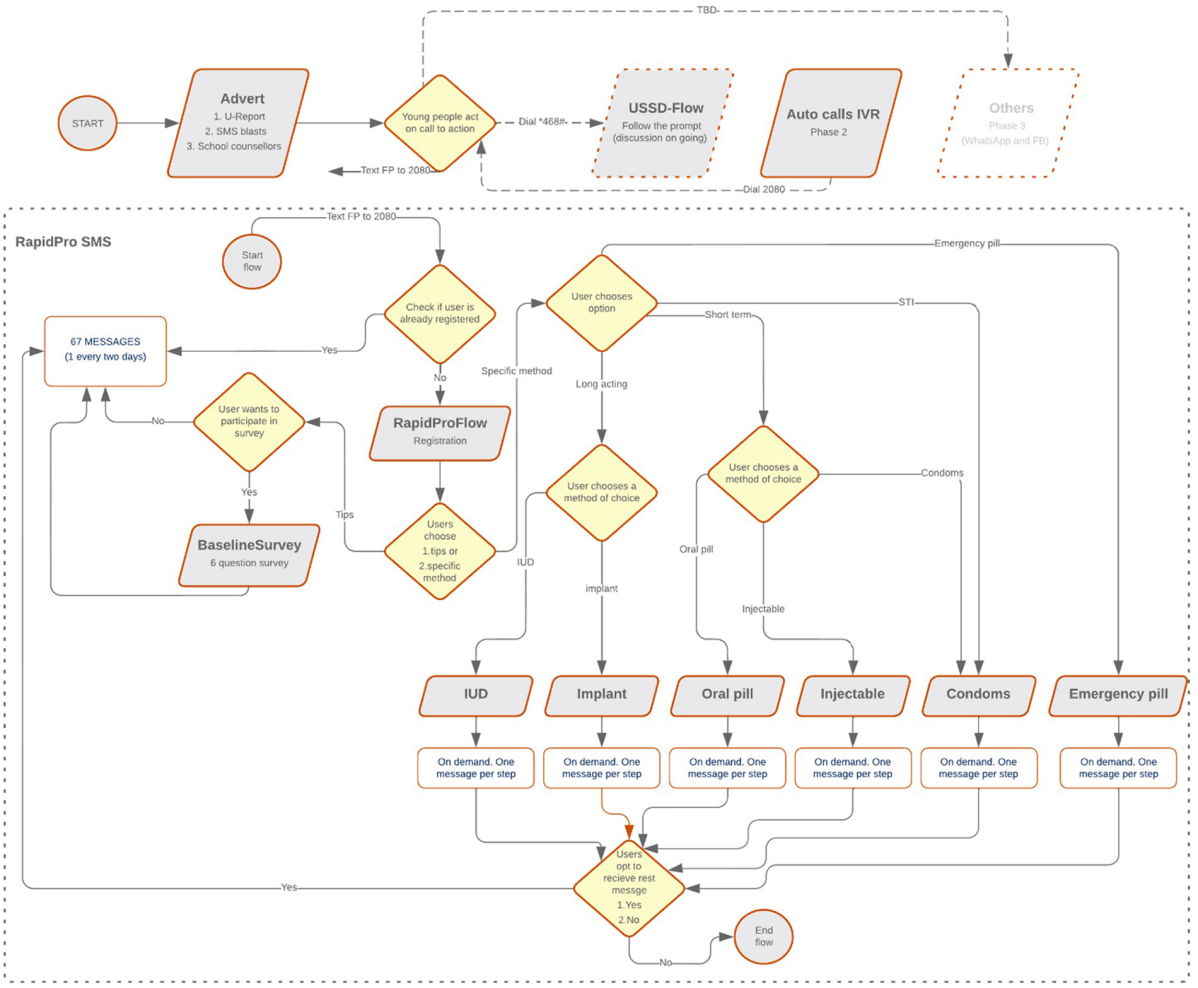
Workflow coded into RapidPro for automated and personalized SMS messaging. SMS, short message service.

In the spirit of One UN, UNFPA in 2019 signed an MoU with UNICEF to use the UNICEF's RapidPro platform to send up to one million interactive SRH SMS messages at no cost to the almost 200,000 registered U-Reporters. Conversely, UNFPA will advertise the 2080 short code to reach 20,000 young people through school counselors, community learning centers, youth advisory panels, social media, and other channels. The message flow was then configured in RapidPro after UNICEF Sierra Leone provided access. The high-level flow for on-demand SRH messages is shown in Figure 4.

##### Collaboration with DSTI

The initial aim was to use the DSTI multi-sectoral platform for the registration of users on the RapidPro platform. As the platform was under development, and the COVID pandemic had limited ability to conduct traditional awareness to drive enrollment and uptake, this option was not used. In addition, according to a recent tweet by the DSTI on 29 September 2022, a milestone 254,669 service usage was recorded on the *468# platform, mainly driven by use for West African Senior School Certificate Exams (WASSCE) results (34).

##### Collaboration with Africell

The GSM Association (GSMA), in their eight-country mNutrition intervention in 2018, collaborated with local telecommunications providers in the project countries (35). There are two main telecommunications service providers in Sierra Leone: Africell and Orange (formerly Airtel). Both have significant telecommunications infrastructure investment and user base. In March 2020, UNFPA reached out to Africell and Orange telecommunications requesting to collaborate on the WiTok mHealth campaign intervention.

Only Africell responded, and subsequently, based on several discussions, an MoU was executed between UNFPA and Africell. Based on the MoU, Africell made available an SMS, and IVR short code (2422), for text and voice content transmission (send and receive) on the Africell network. The MoU required the delivery of SMS to 355,000 subscribers (internet users) and recorded voice messages to 290,000 subscribers (non-Internet users) in the first instance. These numbers were arrived at in discussion with Africell and leveraging the outcome of our 2019 formative research. The 290,000 subscribers represent those covered by the Freetown cell coverage who have never registered for any internet bundle. Similarly, the 355,000 represent the subscribers in Freetown who have subscribed to an internet bundle once in the 90 days before the query.

Africell also offered to make their studio available for recording and provide UNFPA Asterisk server hosting for free. Africell agreed to deliver pre-recorded IVR-style messages to segments (by region or other metrics) or a percentage of their user base. Each voice message will be between 30 s and 1 min. Similarly, each SMS message will be 160 characters or less. UNFPA will pay an agreed lump sum every month for the invoicing service. Africell telecommunications will cover the monthly invoiced costs in excess of this amount. Under the agreement, three calls will be scheduled to 290,000 subscribers per week and three SMS messages to 355,000 subscribers per week. Subsequently, the MoU was extended to deliver three SMS messages per week to all 3.9 million Africell subscribers nationwide.

#### Intervention content of messages deployed to young people

The UNFPA Sierra Leone country office constituted an internal multidisciplinary task team to facilitate the development and adaptation of SRH and GBV content for delivery to Sierra Leoneans. The project approach was different from the traditional interview approach (36). In April 2020, an audiovisual consultant was engaged to support the interpretation and subsequent recording of translated messages from English transcripts to Krio audios for use as telecommunications voice messages and radio messages. A WiTok content team was inaugurated with technical members from MH, FP, and gender-based violence. In the task team were also the communications analyst, the M&E analyst, the audiovisual consultant, and the mHealth consultant. The group started meeting regularly and then extended to online sessions due to the COVID-19 crisis. The team used WhatsApp extensively for online collaborations in April and May 2020.

**Figure 5:**
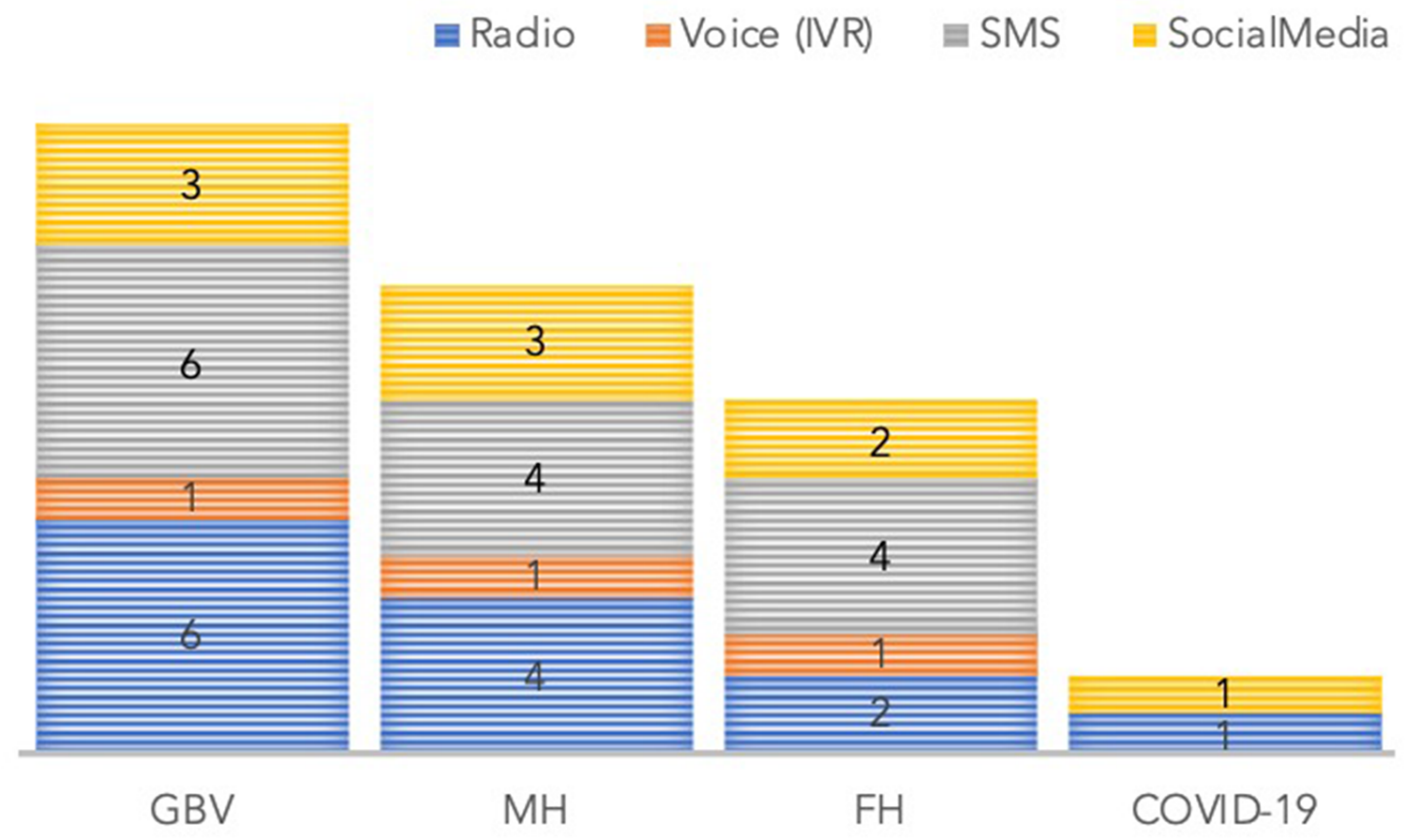
Number of messages by health thematic technical area and delivery channel.

The content team developed English and Krio transcripts of GBV, FP, MH, and COVID-19 messages grouped for radio, telecommunication voice, and SMS (see Appendix 1). The radio messages were recorded in the Krio language, just like the telecommunications voice messages. The content audiovisual consultant working with a team of local experts recorded the messages through an iterative process. The recorded messages had a 1-min time limit and were recorded in a jingle style. The number of messages per health thematic area per delivery channel is illustrated in Figure 5. The voice messages for telecommunication calls were adapted with multimedia content for delivery *via* social media (i.e., Twitter, WhatsApp, and Facebook).

#### Intervention strategy and message delivery schedule

The strategy for content delivery was initially to send messages on GBV, FP, MH, and COVID-19 on alternate days synchronously across all channels in the first phase. However, a limitation in delivering all 290,000 calls a day on the Africell network forced a change in strategy. The strategy for the subsequent week was adjusted to delivering content per health thematic (GBV, MH, FP) area per week to mobile phone users weekly. The first health thematic area was GBV, followed by MH, followed by FP. The messages were targeted to clients based on their network data subscription status. It was untargeted based on lifestyle, gender, education, or economic status. Table 2 details the message schedule for the different health thematic areas and the content delivery channels (37). The messages sent are in Appendix 1.

**Table 2:** Message schedules.

*Week 1 message schedule (Trial week) (April 20–April 26)*				
Day of week	Radio		Voice (IVR)	SMS
Monday	—	—	—
Tuesday	MH, FP, MH, COVID (2 stations)	—	—
Wednesday	MH, FP, MH, COVID	—	MH
Thursday	MH, FP, MH, COVID	MH	—
Friday	MH, FP, MH, COVID	MH	FP
Saturday	MH, FP, MH, COVID	MH	—
Sunday	—	MH	—
*Week 2 schedule for radio, voice calls, and SMS (27th April–3rd May)*				
Day of week	Radio	Voice (IVR)	SMS	Social media
Monday	GBV, FP, MH, COVID	GBV	GBV1	GBV1
Tuesday	GBV, FP, MH, COVID	GBV	—	
Wednesday	GBV, FP, MH, COVID	GBV	GBV5	GBV5
Thursday	GBV, FP, MH, COVID	GBV	—	
Friday	GBV, FP, MH, COVID	GBV	GBV4	GBV4
Saturday	GBV, FP, MH, COVID	GBV	—	
Sunday	—	GBV	—	
*Week 3 schedule for radio, voice calls, and SMS (4th May–10th May)*				
Day of week	Radio	Voice (IVR)	SMS	Social media
Monday	GBV, FP, MH, COVID	MH	MH1	MH1
Tuesday	GBV, FP, MH, COVID	MH	—	—
Wednesday	GBV, FP, MH, COVID	MH	MH2	MH2
Thursday	GBV, FP, MH, COVID	MH	—	—
Friday	GBV, FP, MH, COVID	MH	MH3	MH3
Saturday	GBV, FP, MH, COVID	MH	—	—
Sunday	—	MH	—	—
*Week 4 schedule for radio, voice calls, and SMS (11th May–17th May)*				
Day of week	Radio	Voice (IVR)	SMS	Social media
Monday	GBV, FP, MH, COVID	FP	FP1	FP1
Tuesday	GBV, FP, MH, COVID	FP	—	—
Wednesday	GBV, FP, MH, COVID	FP	FP2	FP2
Thursday	GBV, FP, MH, COVID	FP	—	—
Friday	GBV, FP, MH, COVID	FP	—	—
Saturday	GBV, FP, MH, COVID	FP	—	—
Sunday	—	FP	—	—
*Week 5 schedule for radio, voice calls, and SMS (18th May–22nd May)*				
Day of week	Radio	Voice (IVR)	SMS	Social media
Monday	GBV, FP, MH, COVID	GBV	GBV2	GBV2
Tuesday	GBV, FP, MH, COVID	GBV	—	—
Wednesday	GBV, FP, MH, COVID	GBV	GBV3	GBV3
Thursday	GBV, FP, MH, COVID	GBV	—	—
Friday	GBV, FP, MH, COVID	GBV	GBV6	GBV6
Saturday	GBV, FP, MH, COVID		—	—
Sunday	—		—	—

IVR, interactive voice response; SMS, short message service; GBV, gender-based violence; MH, maternal health; FP, family planning; COVID, coronavirus.

The schedule for radio continued with a mix of all messages daily except Sunday. The aim was that messaging would start the week of 21 April 2020. The proposed schedule is shown in Table 3.

**Table 3:** Four radio stations for disseminating the WiTok recorded contents.

Radio	Morning time	Evening time
Radio Democracy	7:00–7:15 am daily	After 7 pm daily
Africa Youth Voice	10:00–11:00 am daily	6:00–7:00 pm daily
SLBC	9:30–10:00 am daily	8:15–9:00 pm daily
Citizen Radio	7:00–7:15 am daily	7:00–7:15 pm daily

SLBC, SL Broadcasting Cooperation.

### Document review and analysis

The second part of the methodology involved a combination of observational study and case study documentation detailing the methodology and approach leveraged in the deployment of a large-scale messaging intervention. A secondary analysis and process evaluation of project implementation documents and reports aim to understand the design trade-offs and contextual determinants of the successful scaling of a multichannel digital intervention during early stages of COVID-19 pandemic. The list of reviewed documents is shown in Table 4.

**Table 4:** Documents reviewed.

Data Source	Document
UNFPA	mHealth formative research report and article (20)
Africell telecoms	Monthly aggregate intervention message and call logs
UNFPA	Monthly mHealth consultant activity report
116 GBV call center	Monthly news letter
UNFPA	Internal mHealth task team meeting notes

UNFPA, United Nations Populations Fund; GBV, gender-based violence.

According to the WHO classification of digital health intervention version 1, our messaging campaign targeted at potential or current end users of health services in Sierra Leone can be categorized as “intervention for clients” (38). According to the classification, the caregivers of clients who receive service fall within these categories. The other three areas are interventions targeting healthcare professionals, health systems managers, and data services interventions. The intervention whose process is evaluated delivered targeted and untargeted health information to clients in Sierra Leone.

Multiple datasets, including quantitative data extracted from project implementation documents and reports of project evaluation, were repeatedly analyzed and triangulated to facilitate a better understanding of the determinants of the successful scaling of the interventions.

A framework approach was used for the data analysis while allowing for the emergence of new themes. A framework analysis involves the stages of familiarization with data, coding, indexing, charting, mapping, and interpretation (39). Manual data analyses of project implementation documents and the reports of process evaluation were led by EC and SG. All three authors approved the analysis following reviews of extracts to facilitate immersion in the data to identify factors for scaling successful interventions and other contextual factors that shaped project results. Inductive coding was used to understand how the designed messaging interventions improved the health-seeking behavior of recipients (40) and increased the utilization of health services in urban and rural areas. Deductive coding was used identify contextual factors that influenced intervention scale-up.

## Results

### Measuring progress

Usually, messaging interventions are designed to achieve health information promotion and improve the health-seeking behavior of recipients (40). These behaviors often manifest as increased utilization of health services and ultimately lead to improved health outcomes. In this intervention, the main measure was the reach, which was the number of individuals exposed to any of the channels during the messaging campaign. To measure the progress of the project and the reach of messages sent, UNFPA had one of two options to collect the implementation metrics: first, either self-generate the reporting metrics if there was a UNFPA-managed software system; or second, rely on the host organization to generate this information regularly (often monthly). This ability applied to both SMS and IVR/voice calls. Progress metrics were obtained by email from Africell for SMS and voice calls.

**Figure 6:**
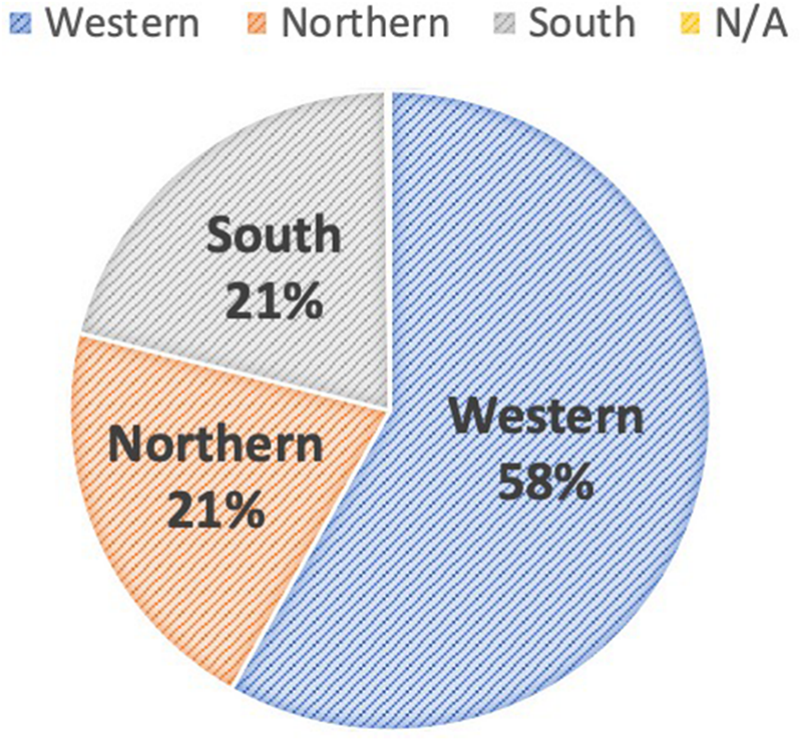
Regional distribution of subscribers who are aged 18–24 years (*n* *=* *2.03 million*).

In all, a total of 1.16 million voice calls were made to 290,000 individuals in Freetown and 34.46 million SMS messages were sent to all 3.9 million Africell subscribers in Sierra Leone. The age distribution of national subscribers shows that 48% did not have an age recorded, and 44% were aged 25 years and above, while only 8% were aged 18–24 years. Figure 6 shows the regional distribution of those who provided an age (52% of 3.9 million); the majority of registered young people aged 18–24 years were in the western region (58%) category.

### Campaign reach

The distribution of reach for both campaign phases is presented in this section by channels.

For radio messages, the number of individuals reached is only determined by estimates from the radio stations themselves. This information will be obtained and made available appropriately. For social media, the primary reach can be obtained from the UNFPA Facebook and Twitter exposure metrics. In addition, the indirect reach through WhatsApp can be estimated by computing the number of members in groups; each message has been shared. WhatsApp was subjective in that this may not translate to actual views.

#### SMS (telecommunications)

**Figure 7:**
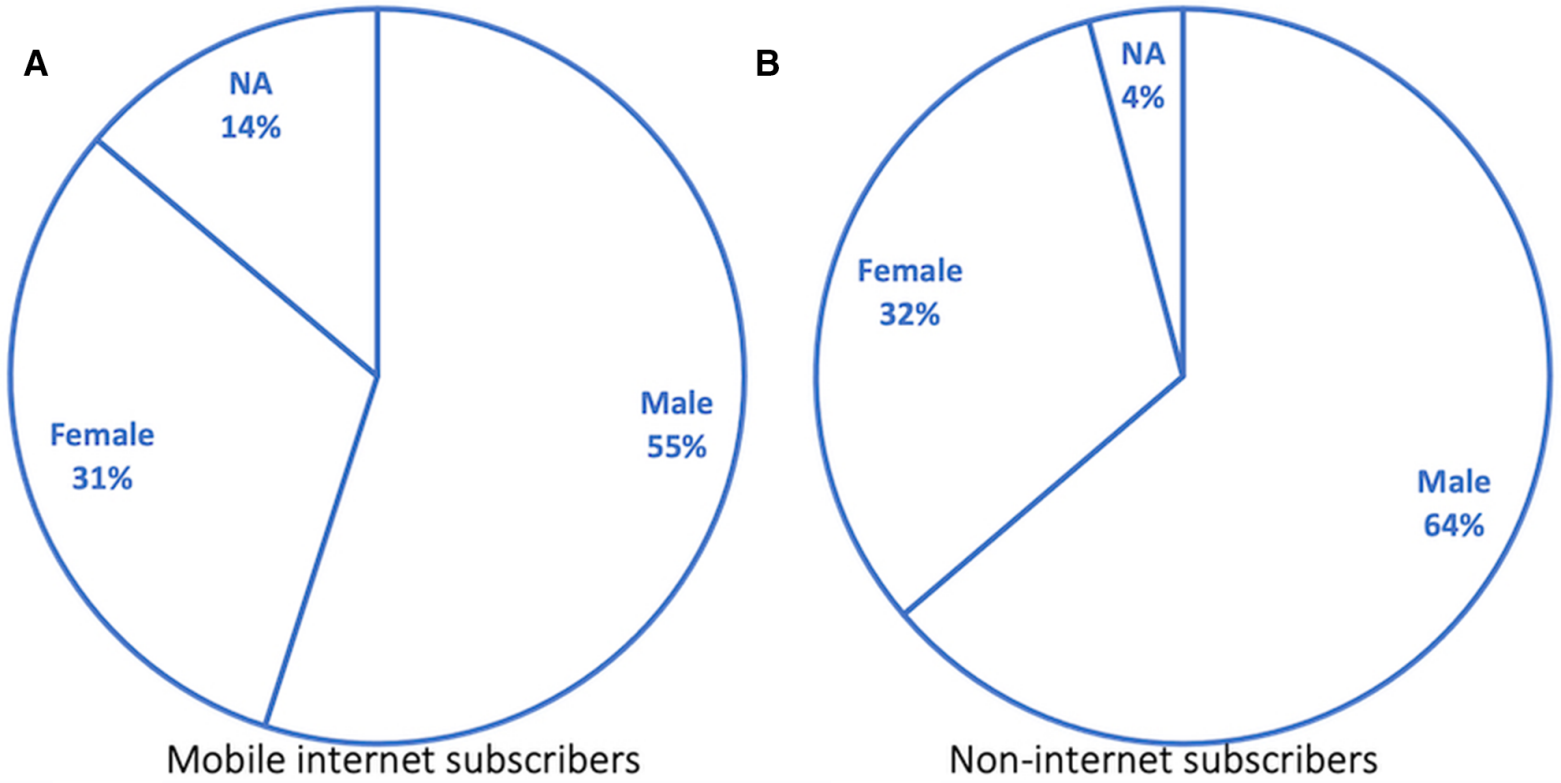
Distribution of targeted subscribers by sex (pilot phase).

The distribution of these subscribers who received SMS in phase one by sex is shown in Figure 7A. In Freetown, 85% of subscribers had provided age data at registration and were aged above 18 years. Only 28% of internet subscribers were aged 18–24 years.

#### Automated calls (telecommunications)

**Figure 8:**
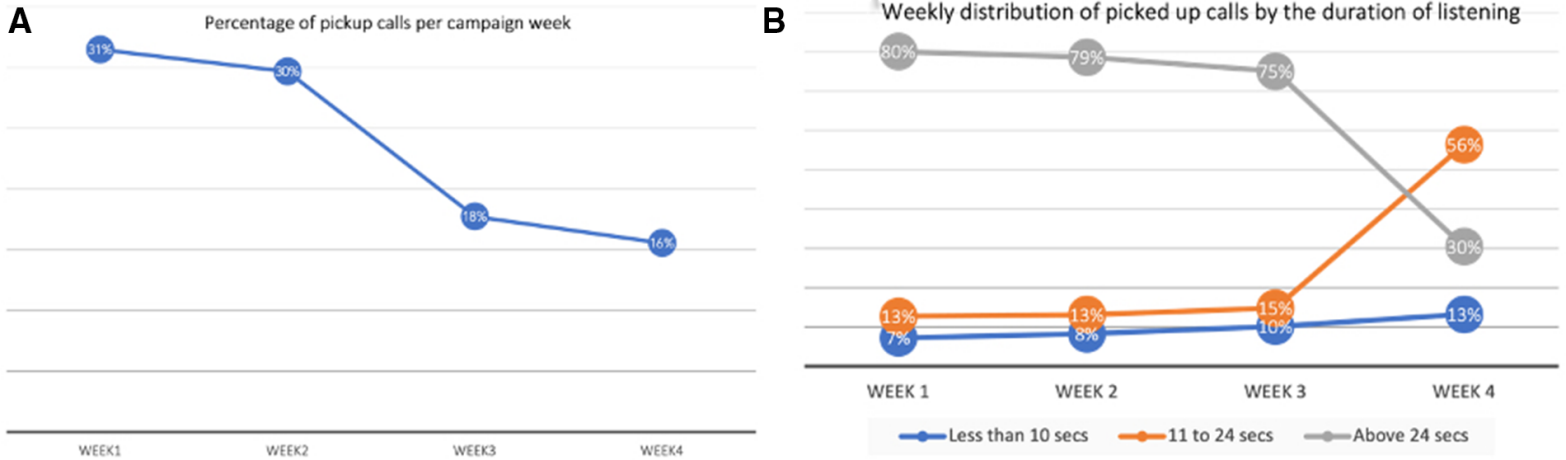
Percentage of call pickups (**A**) and Percentage duration of listening (**B**).

Subscriber segments who have never subscribed to the internet to whom automated calls were initiated and their distribution by sex is shown in Figure 7B. A total of 1.09 million calls were initiated to this subscriber segment. The number of subscribers with age data above 18 years was 80% of total subscribers, while information was not available for 20%. The percentage call pickup rate steadily reduced from 31% to 16% for the campaign's duration, as shown in Figure 8A. See Figure 8B for the distribution of picked-up calls presented by the listening duration. The number of subscribers who did not pick up the 1,093,606 calls was significant, representing 69% in the first week and rising to 84% in the fourth week. Equally, with a 95% confidence level, the drop in the number of persons who picked up the calls from the first week to the fourth week was significant, with a *p* = 1 value. Similarly, those who completely listened to calls dropped significantly from 75% to 30% when the message content was repeated instead of when message content was not repeated (*p* = 1) (Figure 8B).

#### Radio

For both phases of the campaign, the radio campaign reach has been estimated using the population data in Appendix 2 only. In the first phase of the campaign, the four (4) radio stations in Table 4 were used and their schedule is as shown. The radio stations used estimated audience as provided by the stations are in Appendix 2.

#### Social media (Facebook, Twitter, and WhatsApp)

Social media content reach is measured by the number of unique message viewers, while engagement is the number of likes, shares, or comments on Facebook. On Twitter, engagement is the number of likes, retweets, and comments. See details of message distribution in Appendix 3. Each audiovisual message shared to Facebook (FB post), and Twitter (Twits) was also shared to the 39 WhatsApp groups. The corresponding message by thematic area was sent three times each week, on both Twitter and Facebook.

## Discussion, limitations, and lessons

This article highlights a multichannel campaign design and deployment approach while documenting lessons and reach.

### Discussion

#### Telecommunications (SMS and automated calls)

The sex distribution of phone ownership registered to the telecommunications provider shows there were more male phone owners than female owners. This trend is the same for both internet subscribers and non-Internet subscribers (also called 2G subscribers). Due to the socioeconomic distribution of wealth in Sierra Leone, phone sharing is highly likely, and a different subscriber can use another individual's registered SIM card. We established from prior formative research that subscribers who belong to vulnerable groups often use basic phones that are not internet-enabled; these subscribers are also less likely to read and write (20). The formative study also showed that those who cannot read English are less likely to read in any other local dialect (or language) (e.g., Krio). Thus, the nonliterate segment was determined as those without internet and targeted with pre-recorded automated calls in Krio.

We delivered 35.46 million SMS messages over the two phases of the campaign, clearly indicating that SMS is a viable approach for delivering messages. However, we are unable to ascertain how many of these messages were read and understood. A follow-up survey could elicit more information on campaigns like this. We believe this will have the highest impact as SMS messages are retained and can be forwarded and shared with others as needed, though SMS cannot be read by a nonliterate smartphone or internet-enabled phone users. However, it is also possible for nonliterate subscribers to get help from acquaintances to understand these messages.

#### Radio jingles

Our strategy for radio jingles was to help deliver SRH content to mobile-disconnected communities. The actual reach *via* radio campaign remains subjective, as reported by the radio service providers. However, a follow-on survey in Malawi shows that radio is an effective channel for SRH information dissemination (41).

#### Social media

The maximum reach on UNFPA Sierra Leone social media handle was limited by the number of followers. The reach on WhatsApp could not be objectively determined beyond the group membership size. Our measure did not take into consideration potential additional shares and forwards of these messages.

#### Call pickup rates, duration of listening, and overall effectiveness

Registered young people aged 18–24 years represent 12% and 13% of those who picked up calls in the first 2 and last 2 weeks of the campaign, respectively. These numbers are low, considering that this group represents our primary target group for the dissemination of SRH information. However, there was a large percentage of subscribers without age information (48%). Some of these could also be young people. Those that picked up the calls were divided into three groups: those that hung up before 10 s; those that hung up between 11 s and 24 s; and those that completed the message by listening for 25 s or more, which is a 75th percentile average, a measure of completion time of recorded messages.

**Figure 9:**
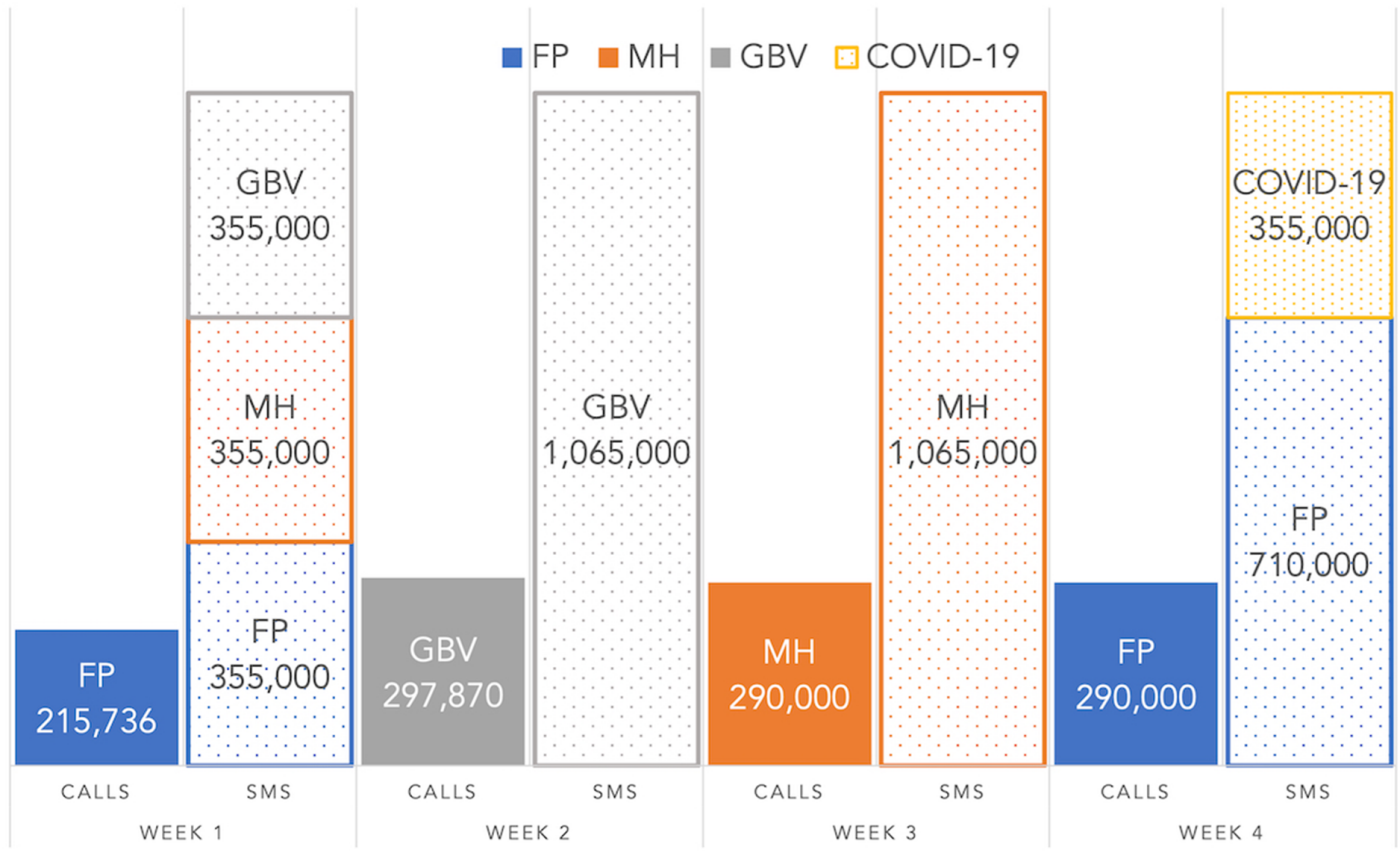
Phase one number of initiated calls and SMS messages per thematic area per week.

The reasons for the different pickup and drop-out durations vary from lack of interest, being busy, to possible network challenges. As the call pickup rate was the highest (at 31%), and the cost per initiated call was about four times the cost of one SMS message, the automated call was not the most viable strategy for a cost-effective campaign. While message repetition for reinforced learning is a proven public health strategy for behavior change, this strategy is not best suited for automated call campaigns. This is demonstrated by the steady drop in the duration of complete listening, as shown in Figure 8B (week 1 – 80%, week 2 – 79%, week 3 – 75%, week 4 – 30%). In the fourth week, the week 1 FP message was repeated as in Figure 9. One strategy to determine the overall effectiveness of the campaign was to measure the service uptake. We used the rate of calls to the newly established 116 helpline to measure this service uptake or request. We acknowledge that other unrelated factors may have affected the uptake of the helpline. However, to our knowledge, no other campaign advertised the 116 helpline in the April campaign period.

### Lessons for future interventions

1. Formative research (20) was crucial to understand the best strategy for content type, targeting strategy, and segment of young people with the most needs.
2. At the time of intervention, short code numbers were issued by MNOs and not NATCOM as earlier envisaged.
3. The limitations of collaboration were evident as strictly following the collaboration strategy would have limited the message reach.
4. The Africell infrastructure could not allow 870,000 concurrent calls every week through their system without significantly degrading their network performance. Implementers should ask for explicit IVR/call and capacity from their telecommunication companies before engaging, including penalty clauses for not reaching the desired call threshold.
5. In addition, neither the SMS nor the IVR platform could be designed and deployed without the technical expertise for making regular tweaks to the system to keep it active and to respond to queries.
6. Collaboration with relevant government programs and telecommunications infrastructure providers were key to content development, approval, and acceptance.
7. Multichannel interventions were critical for reaching different segments of the population, from the nonliterate, through the savvy mobile owners, to the non-mobile-owning community members.
8. Targeted message delivery was found to be difficult to implement, even when done by experienced telecommunications providers.
9. For a messaging campaign, having a list of potential recipients is important for full control; otherwise, partnership with local telecommunication providers will be invaluable, of course at the trade-off of not being able to target initially per user needs.
10. The language of the recipient, as seen from the formative study, is an important determinant in the choice of message structure and delivery channel.
11. The choice of delivering messages that the user can engage with (two-way) versus a one-way message (push-only) was made with significant trade-offs. Push-only can reach more people, while two-way can only reach a few individuals. This lesson is particularly important in the context of the speed needed during the pandemic.
12. The choice of channels, like voice calls, while able to reach more nonliterate users, is prohibitively expensive and should only be used minimally or in a situation where cost is not a consideration.
13. When negotiating the cost of a recorded voice message, a clear distinction needs to be made between the price for initiated calls and the price for listened calls, as only 30% of calls were actually picked up.
14. Pre-recorded calls were costed as “initiated calls” and not as “picked-up calls” rates. Hence, cost charges apply even when subscribers do not pick up the calls. This makes the use of automated calls as a campaign strategy expensive. Each voice call is more than four times more expensive than the cost per SMS, yet only 31% of all initiated calls were picked up.
15. A key lesson is that important messages must appear in the first few seconds of a call or IVR campaign. In addition, an average of 80% of picked-up calls were entirely listened to (24 s or more) except for when the recorded message was repeated (see Figure 8B, 3). When the FP call was repeated in week 4, the number of picked-up calls that listened for 24 s or more dropped to 30% from 80%.
16. Targeting by sex, age group, data usage, and location is difficult for mobile phone subscribers as 48% of subscribers had missing information regarding age, sex, or location. In addition, for subscribers where such information was available, it was unreliable as sometimes people subscribe on behalf of others, particularly those aged under 18 years.
17. Radio stations can help reach nonliterate users in their local language, though effective reach and measurement of impact remain subjective. Social media (Facebook and Twitter) can be used to target page followers or social media users by age, sex, location, and other user interests through platform user metrics.
18. When telecommunications providers deliver content to their subscriber base, there is a limit to available data metrics. Call logs may only be available in aggregate formats, which cannot be independently verified. Radio jingles can be tracked per play per radio station, but their reach is much more challenging to measure. Facebook and Twitter reach metrics provide more reliable details of reach, clicks, likes, and shares. However, the audience is limited to literate subscribers.
19. Other factors, such as listening duration, should be considered when delivering non-targeted recorded voice messages.

### Limitations

Given that many subscribers (48%) did not provide age information, it was difficult to deduce the complete number of young people (18–24 years) reached by the telecommunications campaign. The current figures are likely only a fraction of young people reached. In addition, since there was no way to determine whether an SMS gets delivered or has been read by the subscriber, impact measure would require more activities beyond the scope of this paper. In addition, the study did not evaluate the potential impact of service uptake at health facilities or the potential reduction to GBV violence as a result of the availability of the helpline or of receiving the messages.

This paper used the self-reported radio campaign coverage, which is the population of the respective district. This is subjective as not everyone has a radio, and not all age groups listen to the radio; hence, we believe the actual listenership is far lower than the population coverage quoted. For automated calls, we are not able to determine the exact reason for the given duration of listening. Though the intervention was a one-time intervention, which has now ceased, its aim was to ensure that Sierra Leoneans were aware of continuity of service during a pandemic.

## Conclusion

In this work, the experience of designing and implementing WiTok, a large-scale mobile SRH messaging campaign in Sierra Leone, is described. The design considerations and lessons from the deployment through multiple channels are discussed. These are, notably, the impact of a formative research to large-scale messaging solutions, intervention deployment, technology used, organization technology capacity, and cost considerations. In addition, there were trade-offs between two-way messaging and push-only messaging regarding reach.

A total of 1.09 million calls were initiated to 290,000 subscribers in Freetown, Sierra Leone. Similarly, 35.46 million SMS messages were sent out to 3.9 million subscribers nationwide in Sierra Leone. In both phases, SRH and GBV messages were aired at all 15 radio stations across the country. The maximum exposure (reach) on either UNFPA's Facebook or Twitter page was 4,910. Audiovisual messages were shared in at least 39 Sierra Leone WhatsApp groups.

There were twice as many male subscribers than female subscribers in Freetown, and this is nearly so nationally. The call pickup rate was 31% in the first week and dropped to 16% in the fourth week of the automated call campaign. Among those who picked up the call, those who listened for 24 s or more dropped from 75% to 30% when the message was repeated. The first campaign targeted 645,000 Africell subscribers in Freetown, in addition to those reached by four Freetown radio stations and social media. The national campaign (second campaign) targeted 3.9 million subscribers representing all active subscribers on the Africell network in Sierra Leone.

The difficulty in measuring reach was equally highlighted. During the intervention period, there was an increase in the national GBV calls received *via* the 116 call center helpline. While improvements may have been recorded, they may not be attributable to this multichannel messaging intervention campaign alone due to multiple projects implemented at the peak of the pandemic in Sierra Leone. Other implementing organizations had competing interventions to address similar project objectives.

## Data Availability

The original contributions presented in the study are included in the article/Supplementary Material, further inquiries can be directed to the corresponding author.

## APPENDIX 1 SMS messages sent to subscribers in Freetown (phase one) and Nationally (phase two).

**Table:**

	(a) Phase one (20th April 2020 to 22nd May 2020)
	Health (GBV, MH, FP) messages delivered to target beneficiaries *via* SMS in Freetown (Trial week 1)
1	Are you experiencing violence? Violence can be sexual, physical, or emotional and can happen to you! Even in your own home! Don't delay, Call 116 for help!
2	Are you pregnant and bleeding? Have stomach pains, headache, or fever? Don't delay, visit the clinic! Don't let fear of Corona stop you, a midwife can help you
3	Use family planning if you don't want to get pregnant, it is free at government clinics and hospitals. Don't be afraid of Corona, go to your nearest clinic
	GBV messages delivered to target beneficiaries *via* SMS in Freetown (Week 2)
1	Are you experiencing violence? Violence can be sexual, physical or emotional and can happen to you! Even in your own home! Don't delay, Call 116 for help!
2	Violence at home is never ok. Never use physical, sexual or emotional violence in the home. If you experience violence at home, go to FSU or call 116 for help!
3	Women and girls are at increased risk of sexual, physical or emotional violence. Help to protect the rights of women, girls especially those with disabilities!
	MH messages delivered to target beneficiaries *via* SMS in Freetown (Week 3)
1	Go for ANC as soon as you get pregnant and attend all ANC visits. The midwife can help you look after yourself and your baby.
2	Free healthcare is for pregnant women and breastfeeding mothers. If you are pregnant or just had a baby, visit your nearest clinic for free healthcare.
3	Giving birth at home can lead to serious complications for you and your baby, you can even die. Deliver your baby at a clinic with a midwife. Don't fear Corona
	FP and COVID messages delivered to target beneficiaries *via* SMS in Freetown (Week 4)
1	Use family planning if you don't want to get pregnant, it is free at government clinics and hospitals. Don't be afraid of Corona, go to your nearest clinic
2	Condoms protect you from pregnancy, HIV and other Sexually Transmitted Infections (STIs). Even with coronavirus you can still visit hospitals if are pregnant or to join family planning
3	To prevent corona virus, avoid touching your face, cover your nose and mouth when you cough or sneeze, wash your hands regularly with soap and water.
	
	(b) Phase two (3rd July 2020 to 26th July 2020)
	GBV messages delivered to target beneficiaries *via* SMS nationwide (Week 1)
1	No one has the right to force you for sex. EVERYONE has the right to say NO! Rape is a crime! If it happens to you, don't delay, go to FSU or call 116 for help!
2	It is against the law to have sex with or to marry a child under 18 years, it is a crime. Protect our children and report offenses!
	GBV messages delivered to target beneficiaries *via* SMS nationwide (Week 2)
1	Women and girls are at increased risk of sexual, physical or emotional violence. Help to protect the rights of women, girls especially those with disabilities!
2	If you try to settle a sexual offense such as rape out of court, you will be convicted and or fined. Such a compromise is a crime!
	GBV messages delivered to target beneficiaries *via* SMS nationwide (Week 3)
1	No one has the right to force you for sex. EVERYONE has the right to say NO! Rape is a crime! If it happens to you, don't delay, go to FSU or call 116 for help!
2	It is against the law to have sex with or to marry a child under 18 years, it is a crime. Protect our children and report offenses!
	GBV messages delivered to target beneficiaries *via* SMS nationwide (Week 4)
1	Women and girls are at increased risk of sexual, physical or emotional violence. Help to protect the rights of women, girls especially those with disabilities!
2	If you try to settle a sexual offense such as rape out of court, you will be convicted and or fined. Such a compromise is a crime!

## APPENDIX 2 The list and distribution of radio stations used for jingle dissemination.

**Table:**

District	Radio station	Covered population
1. Bo	Kiss 104	575,478
2. Bonthe	Radio Bontico	200,781
3. Moyamba	Radio Modcar	318,588
4. Pujehun	Radio Wanjei	346,461
5. Tonkolili	Radio Gbafth	531,435
6. Makeni	Hope FM	606,544
7. Koinadugu	Radio Bintumani	409,372
8. Port Loko	Radio Gbankasoka	615,376
9. Kambia	Radio Kolenten	345,474
10. Kailahun	SL Broadcasting Cooperation (SLBC)	526,379
11. Kenema	Radio Nongowa	609,891
12. Kono	Eastern Radio	506,100
13. Western Rural	Voice of the Peninsula	444,270
14. Freetown (Western Urban)	SL Broadcasting Cooperation	1,055,964
15. Freetown (Western Urban)	Africa Young Voice	1,055,964

## APPENDIX 3 The number of Twitter and Facebook reach, engagement, and shares per week.

**Table:**

	Week 1 (mixed themes)			Week 2 (GBV)			Week 3 (MH)			Week 4 (FP and COVID-19)		
	Reach (views)	Engagement (Like, comment)	Retweets or Shares	Reach	Engagement	Retweets or Shares	Reach	Engagement	Retweets or Shares	Reach	Engagement	Retweets or Shares
Twit 1	2,583	119	14	3224	156	15	2385	76	13	4910	246	13
Twit 2	1,950	85	11	2004	77	5	2324	46	8	1500	104	10
Twit 3	2689	108	14	1253	32	7	4910	246	13	2105	111	9
												
FB post 1	656	27	5	369	24	1	934	23	4	261	22	0
FB post 2	1,366	62	5	216	7	0	1770	188	4	337	30	0
FB post 3	843	29	1	226	8	1	295	12	0	295	33	1
